# Reliable detection of subchromosomal deletions and duplications using cell‐based noninvasive prenatal testing

**DOI:** 10.1002/pd.5377

**Published:** 2018-11-19

**Authors:** Liesbeth Vossaert, Qun Wang, Roseen Salman, Xinming Zhuo, Chunjing Qu, David Henke, Ron Seubert, Jennifer Chow, Lance U'ren, Brennan Enright, Jackie Stilwell, Eric Kaldjian, Yaping Yang, Chad Shaw, Brynn Levy, Ronald Wapner, Amy Breman, Ignatia Van den Veyver, Arthur Beaudet

**Affiliations:** ^1^ Department of Molecular and Human Genetics Baylor College of Medicine Houston TX USA; ^2^ Baylor Genetics Laboratory Houston TX USA; ^3^ RareCyte Inc. Seattle WA USA; ^4^ Immune Design Seattle WA USA; ^5^ Department of Pathology and Cell Biology Columbia University Medical Center New York NY USA; ^6^ Department of Obstetrics and Gynecology Columbia University Medical Center New York NY USA; ^7^ Department of Obstetrics and Gynecology Baylor College of Medicine Houston TX USA

## Abstract

**Objective:**

To gather additional data on the ability to detect subchromosomal abnormalities of various sizes in single fetal cells isolated from maternal blood, using low‐coverage shotgun next‐generation sequencing for cell‐based noninvasive prenatal testing (NIPT).

**Method:**

Fetal trophoblasts were recovered from approximately 30 mL of maternal blood using maternal white blood cell depletion, density‐based cell separation, immunofluorescence staining, and high‐resolution scanning. These trophoblastic cells were picked as single cells and underwent whole genome amplification for subsequent genome‐wide copy number analysis and genotyping to confirm the fetal origin of the cells.

**Results:**

Applying our fetal cell isolation method to a series of 125 maternal blood samples, we detected on average 4.17 putative fetal cells/sample. The series included 15 cases with clinically diagnosed fetal aneuploidies and five cases with subchromosomal abnormalities. This method was capable of detecting findings that were 1 to 2 Mb in size, and all were concordant with the microarray or karyotype data obtained on a fetal sample. A minority of fetal cells showed evidence of genome degradation likely related to apoptosis.

**Conclusion:**

We demonstrate that this cell‐based NIPT method has the capacity to reliably diagnose fetal chromosomal abnormalities down to 1 to 2 Mb in size.

## Abstract 2

What is already known about this topic?

- Fetal trophoblastic cells can be isolated from maternal blood and be used for the detection of fetal aneuploidies and copy number variants. The data on the detection of subchromosomal deletions and duplications is currently limited.

What does this study add?

- Cell‐based NIPT can be used for the detection of copy number abnormalities of greater than or equal to 1 Mb in the fetus by low‐coverage next‐generation sequencing after single cell whole genome amplification. Data are provided here for five cases in which different subchromosomal deletions and duplications ranging from 1.2 to 18.9 Mb were detected in single cells.

**What is already known about this topic?**

- Fetal trophoblastic cells can be isolated from maternal blood and be used for the detection of fetal aneuploidies and copy number variants. The data on the detection of subchromosomal delestions and duplications is currently limited.

**What does this study add?**

- Cell‐based NIPT can be used for the detection of copy number abnormalities of greater than or equal to 1 Mb in the fetus by low‐coverage next‐generation sequencing after single cell whole genome amplification. Data are provided here for five cases in which different subchromosomal deletions and duplications ranging from 1.2 to 18.9 Mb were detected in single cells.

## 1 INTRODUCTION

In recent years, the field of prenatal testing has been transformed with the clinical implementation of cell‐free DNA (cfDNA)‐based analysis, known as noninvasive prenatal testing (NIPT). Despite its clearly higher positive predictive value for trisomy 21 compared with traditional first trimester serum analyte screening for both low‐risk and high‐risk pregnancies, the test's performance is well below that of diagnostic methods, and confirmatory testing is important for all women with positive NIPT results, especially for subchromosomal copy number variants (CNVs). cfDNA‐based NIPT is currently only recommended for common fetal aneuploidies but not for screening for microdeletions/duplications in statements from professional societies.^1^, ^2^ During a normal pregnancy, only 5% to 20% of the total cfDNA pool is of fetal origin, referred to as the fetal fraction.^3^ The current NIPT methodology thus relies on identifying a chromosomal abnormality in an amalgamation of maternal and fetal DNA fragments, which can lead to false positive results, and its performance can be affected by a below average fetal fraction (<4%). cfDNA‐based NIPT is also potentially influenced by maternal chromosomal mosaicism or maternal malignancies.^4^ It thus remains a screening test requiring diagnostic testing for confirmation of positive results. Since the clinical implementation of cfDNA‐based NIPT, the number of Chorionic villus sampling (CVS)/amniocentesis procedures performed has decreased substantially over recent years.^5^, ^6^, ^7^ While this reduces the procedure‐related risk for pregnancy loss, it also leads to failure to diagnose clinically significant subchromosomal abnormalities such as deletion and duplication syndromes, easily detectable with chromosomal microarray (CMA), the current standard diagnostic test of DNA extracted from amniotic fluid or chorionic villi.

In contrast, cell‐based NIPT offers a more attractive alternative if it can be performed reproducibly and at reasonable cost. Although cell‐based NIPT also has limitations such as the risk of too few cells recovered, the specific isolation of multiple individual fetal cells from the maternal circulation offers the advantage of providing pure fetal DNA, free of maternal DNA contamination. As such, the fetal genome can be analyzed at a higher resolution, allowing for the detection of CNVs as small as 1 to 2 Mb in size. This would thus allow for increased accuracy and improved positive and negative predictive values compared with cfDNA‐based NIPT in detecting microdeletion syndromes that are responsible for a range of rare conditions including some cases of autism and intellectual disability and can be detected in up to 1.7% of amniotic fluid or CVS samples from pregnancies without fetal anomalies.^8^ Additionally, the analysis of multiple individual fetal cells from one sample yields data replicates, creating the potential for a higher test result confidence and to identify two different fetal genotypes in case of confined placental mosaicism.

Multiple recent publications^9^, ^10^, ^11^, ^12^ substantiate the feasibility of this approach and show concordant results with the corresponding microarray and karyotype data from invasive diagnostic testing, including a case in which the fetus was affected with a 2.7‐Mb deletion of chromosome 15.^9^ This was the first indication that cell‐based NIPT has the capacity to perform at the resolution required for a clinically diagnostic prenatal test. The published methods describe the enrichment of fetal trophoblastic cells, by either depletion of maternal cells^9^ or specific fetal cell positive enrichment,^10^, ^11^, ^12^ for downstream genome‐wide CNV analysis. These publications, along with earlier reports of a high failure rate when attempting to isolate fetal nucleated red blood cells (fnRBCs),^13^ and our own unpublished failed fnRBC attempts guided our decision to focus initially on circulating trophoblasts. Fetal nRBCs would certainly be attractive and avoid confined placental mosaicism, if successful and consistent recovery and analysis can be achieved, but this has not been demonstrated so far. In this follow‐up report, we present multiple additional cases processed with the aforementioned depletion protocol. In this method, fetal trophoblasts are isolated and analyzed, after depletion of maternal white blood cells (WBCs), immunostaining, high‐resolution scanning, visual verification of target cells, subsequent whole genome amplification (WGA) and low coverage (0.3‐0.6X, 100 bp paired‐end reads) single cell next‐generation sequencing (NGS).

## 2 METHODS

### 2.1 Sample collection

Blood samples from pregnant women were collected after informed consent, under a protocol approved by the Institutional Review Boards of Baylor College of Medicine and Columbia University. The study subjects were recruited following routine prenatal genetic counseling, and in many cases also underwent CVS or amniocentesis followed by conventional chromosome analysis and/or CMA. Approximately 30 mL maternal venous blood was drawn into blood collection tubes containing a proprietary preservative (RareCyte) for trophoblast enrichment. An additional 4 mL was collected in Ethylene‐Diamine‐Tetra‐acetic acid (EDTA) tubes for maternal genomic DNA (gDNA) extraction and fetal cfDNA collection for fetal sex determination. When possible, a 2‐mL blood sample in EDTA or saliva (Oragene) from the father was also collected. The tubes for fetal cell isolation were kept overnight at room temperature or shipped by overnight carrier at ambient temperature until further processing the next day. Maternal gDNA extraction (and paternal when available), fetal cfDNA extraction, and quantitative polymerase chain reaction (PCR) for fetal sex determination were performed as previously described.^9^ Table 1 summarizes this sample series' characteristics, more detailed information is available in the Table S1.

**Table 1:** Sample series characteristics

**125 Blood Samples** Collected from **122 Pregnant Women** (Including Three Redraws)			
**Plurality**	Singleton	118 pregnancies	96.7%
	Twin	4 pregnancies	3.3%
**Fetal sex**	Female	53 pregnancies	43.4%
	Male	65 pregnancies	53.3%
	Twin – F + F	1 pregnancy	0.8%
	Twin – F + M	2 pregnancies	1.6%
	Twin – M + M	1 pregnancy	0.8%
**Maternal age**	Range	19‐41 y/o	
	Median	32 y/o	
**Maternal BMI**	Range	18.90‐45.89 kg/m^2^	
	Median	25.34 kg/m^2^	
**GA at sampling**	Range	10 weeks and 2 days to 35 weeks and 1 day	
	Median	12 weeks and 6 days	
**Recruitment**	Houston	87 samples	69.6%
	New York	38 samples	30.4%
**Paternal samples**	Not available for	71 pregnancies	58.2%
	Available for	51 pregnancies	41.8%
		Blood: 44 samples (84.8%)	
		Saliva: 7 samples (15.2%)	
**Diagnostic testing**	No testing	44 women	36.1%
	CVS	42 women *(*4 samples PP*)*	34.4%
	Amniocentesis	33 women *(*3 samples PP*)*	27.0%
	Both	3 women	2.5%

Abbreviations: F, female; M, male; GA, gestation age; PP, postprocedure (= blood sample collected few minutes to 2 hours after diagnostic procedure). Of note, BMI data were only available for 103 out of 125 samples (=82.4%).

### 2.2 Trophoblast enrichment and isolation

Trophoblasts were enriched and stained as previously described,^9^ with the inclusion of a maternal WBC depletion step for all samples. The amount of depleted maternal WBCs ranged from 79.6% to 99.3%, with an average of 91.5% based on cell counting. After the depletion step, all nucleated cells were separated based on density centrifugation, fixed, and stained. All samples were spread on CyteSlides (RareCyte, 800 000 cells/well) and scanned on the CyteFinder instrument (RareCyte), as previously described.^9^ All putative trophoblasts meeting the internally specified criteria (specific nuclear morphology, cytokeratin‐positive staining in a defined pattern, and WBC marker CD45‐negative) were picked as single cells and deposited in 2 uL of PBS in a PCR tube and stored at −80°C until further processing.

We applied this protocol to 125 samples from 122 women (three of whom had two blood draws), including 15 cases with a fetal aneuploidy and five in which a fetal subchromosomal abnormality was diagnosed.

### 2.3 Whole genome amplification and genotyping

Single cells were processed with the PicoPLEX WGA kit (Rubicon, now Takara Bio) as previously described.^9^ All purified WGA products were stored at −20°C until further use.

A genotyping assay was developed for the confirmation of fetal origin of the isolated fetal cell candidates (Zhuo et al manuscript in preparation). With this NGS‐based assay, a series of informative single‐nucleotide polymorphisms (SNPs) in the trophoblast WGA products are analyzed and compared with the SNP profile of the maternal and when available paternal gDNA. A cell is scored as being fetal when the corresponding WGA products show two or more polymorphic alleles not present in the maternal gDNA; one such allele is scored as likely fetal. Based on data from male pregnancies, the prior probability that a female cell from a female pregnancy is fetal is 89%, and using a Bayesian calculation, the presence of one or two nonmaternal alleles gives a very high probability that the cell is fetal.

### 2.4 CNV analysis

Sequencing for genome‐wide copy number detection and subsequent analysis was done as previously described.^9^ In short, library preparation was started from 300 ng of WGA product after which paired‐end, whole genome sequencing was performed on a HiSeq platform (Illumina), aiming for 5 to 10 x 10^6^ unique reads per cell (100 bp read length), giving a genome coverage of about 0.3 to 0.6X. An in house‐developed web tool was used to generate a view with both a whole genome (1 Mb bin size) and detailed single chromosome plots (100 kb bins). Similar results are obtained with other commercially available tools (eg, BioDiscovery Inc.). CNV analysis was not done for all cases.

A more detailed methods description is provided in the Data S1.

## 3 RESULTS

### 3.1 Fetal trophoblast yield

A range of 0 to 38 trophoblastic cells per sample was identified by microscopy, corresponding to an average of 4.17 cells/sample or 0.18 cells/mL of maternal blood (Table 2). For the four twin pregnancy samples, 12, 9, 14, and 15 trophoblastic cells were found, corresponding to 0.50, 0.50, 0.58, and 0.63 cells/mL respectively. For 27 samples, some fetal cells appeared in a cluster of two to five cells (Table S1). These grouped cells were mostly picked as one cluster and also analyzed as such. All other trophoblasts undergoing CNV assessment were analyzed as single cells.

**Table 2:** Fetal trophoblast yield

**Overview Fetal Trophoblast Identification**		
**Average cells identified by microscopy**	4.17 cells/sample (range 0‐38 cells/sample)	
	0.18 cells/mL maternal blood (range 0‐1.58 cells/mL)	
**Trophoblast distribution** (total of 125 samples collected)		
0 cells	23 samples	18.4%
1 cells	24 samples	19.2%
2 cells	11 samples	8.8%
3 or 4 cells	23 samples	18.4%
5 cells or more	44 samples	35.2%

For male pregnancies (confirmed by clinical information and fetal sex determination based on cfDNA PCR), the fetal origin of the cells can be assessed by NGS analysis of X and Y copy number. Out of the 103 male putative trophoblasts analyzed, 11 showed an XX complement instead of XY and were thus of likely maternal origin. For female fetuses and twin pregnancies, 188 cells out of 289 putative cells were genotyped, and the fetal or likely fetal origin was confirmed for 56.4% and 6.4%, respectively. The genotyping result was uninformative or the assay failed for 35.1%. Four cells were confirmed maternal.

In total, 38 samples out of 125 were shipped overnight from New York to Houston, versus 87 samples recruited locally. On average 0.16 cells/mL were found for the first group compared with 0.18 cells/mL for the latter; this difference was not statistically significant (*P* value: 0.616; unpaired *t* test, two tailed), showing that overnight shipment did not pose a problem for trophoblast recovery.

In total, there were seven samples that were collected within a few minutes to 2 hours after CVS or amniocentesis procedure. Within this group, fetal cells were identified only in four samples: only one cell was found for one post CVS and one postamnio sample, while two other post CVS samples had 9 and 12 cells, respectively.

We investigated whether the maternal body mass index (BMI) had an influence on the fetal cell yield (cells/mL). BMI data was available for 103 samples and ranged from 18.90 to 45.89 kg/m^2^. No significant correlation was found (Pearson r: 0.065; *P* value: 0.513). Although we observed fewer trophoblasts with increasing gestational age, also here, no significant correlation was found (Pearson r: −0.144; *P* value: 0.112).

### 3.2 Abnormal findings: Fetal aneuploidies

This sample series contained multiple cases with chromosomal abnormalities as detected by diagnostic prenatal testing. There were 15 cases with clinically reported fetal aneuploidies, including eight fetuses with trisomy 21 (of which two were the result of a Robertsonian translocation), four with trisomy 18, two cases with 45,X without mosaicism, and one case with 47,XXY. We did not recover any fetal cells for one of the translocation Down syndrome cases, two of the trisomy 18 cases, and the 47,XXY case, but we found on average 4.27 cells/sample for the other 11 aneuploidy cases (0.18 cells/mL), all collected between 12 and 18 weeks gestation. For all of these, the cell‐based NIPT results were consistent with the diagnostic results.

**Figure 1:**
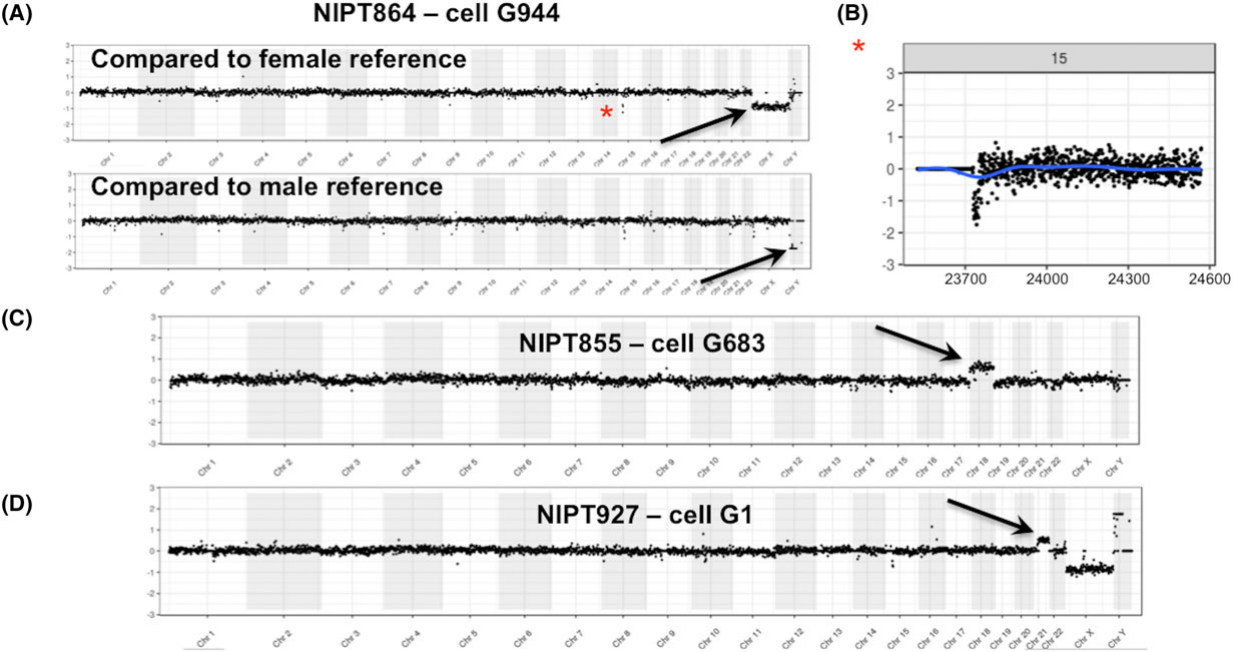
Detection of fetal aneuploidies. Panel A shows the single cell next‐generation sequencing (NGS) analysis on three cells from a pregnancy in which the fetus had a 45,X chromosome complement. The whole genome plots show the comparison of the fetal cells to normal fetal reference single cells. In Panel A, three cells are compared with a single normal female fetal cell, and the loss of X is visible (arrows), with no change in Y. In Panel B, the same fetal cells are compared with a male reference showing a loss of Y (arrows) but no change for X. A copy number loss in the case versus the references in the polymorphic region of 15q11 can be seen in all plots (asterisk). A more detailed view of chr15 is shown under the whole genome plots (comparison to female reference). Panel C shows a female fetal cell with trisomy 18 and Panel D a male fetal cell with trisomy 21. For all figures, the NGS whole genome plots are displayed as 1 Mb bins and the detailed single chromosome plots as 100 kb bins. Additional plots for each of these cases are shown in Figure **S1** [Colour figure can be viewed at wileyonlinelibrary.com]

Panel A in Figure 1 shows one of the 45,X cases. This subject underwent a CVS at 14 weeks and 2 days gestation, after ultrasound examination showed fetal cystic hygroma, hydrops, pleural effusion, echogenic bowel, and a nuchal translucency (NT) of 14 mm. Analysis of the chorionic villus sample indicated a 45,X karyotype, as was also seen in the sequencing data from three single fetal trophoblasts isolated from a maternal blood sample obtained before the CVS procedure was done. Two additional aneuploidy cases are shown in Panels C and D: one female fetus with trisomy 18 and one male fetus with trisomy 21, respectively. Additional data on each of these cases are shown in Figure **S1**.

### 3.3 Abnormal findings: Subchromosomal variants

**Figure 2:**
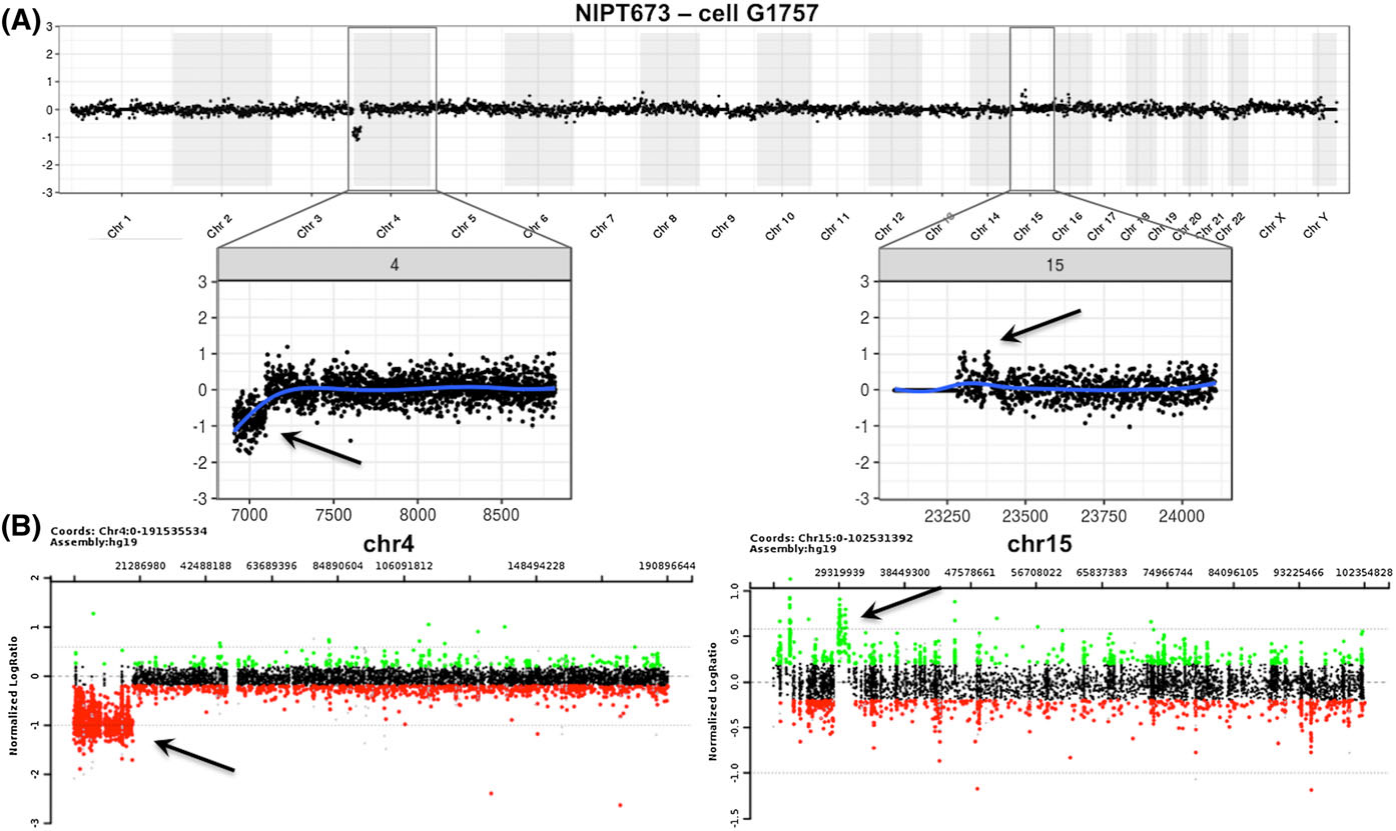
Detection of an 18.9 Mb deletion on chromosome 4p and a smaller duplication on chromosome 15q in a female fetus. The cell‐based noninvasive prenatal testing (NIPT) result is shown in Panel A and the amniocentesis result in Panel B. Chromosomal microarray (CMA) after amniocentesis revealed an 18.9‐Mb deletion of 4p16.3p15.31 (Wolf‐Hirschhorn region) and a 1.1‐Mb gain of 15q13.1q13.2. The CMA coordinates for the deletion are chr4:85,743‐18,953,893 and for the gain chr15:29,213,743‐30,300,265. The cell‐based NIPT data were concordant with these amnio data [Colour figure can be viewed at wileyonlinelibrary.com]

**Figure 3:**
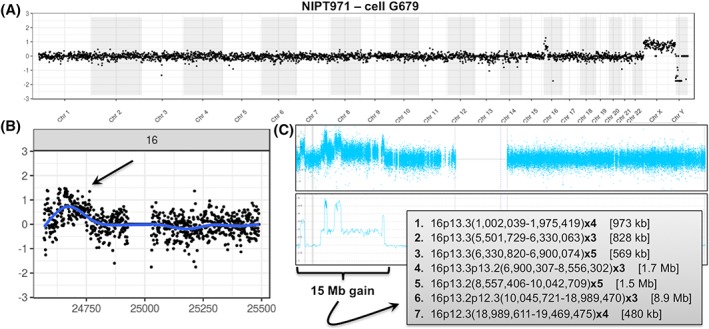
Detection of a complex subchromosomal gain on chromosome 16p. Panels A and B show the cell‐based noninvasive prenatal testing (NIPT) whole genome and chromosome 16 plot, respectively, of the comparison of a female fetal cell compared with a normal male reference. A gain is seen on chromosome 16p. A single‐nucleotide polymorphisms (SNP) array comparative genomic hybridization (CGH) on a chorionic villus sampling (CVS) sample as shown in Panel C, indicated a large complex 15 Mb gain of chromosome 16, with three duplications, three triplications, and two quadruplications. The details of the subsections of the gain detected on array cannot be distinguished in the cell‐based NIPT data [Colour figure can be viewed at wileyonlinelibrary.com]

**Figure 4:**
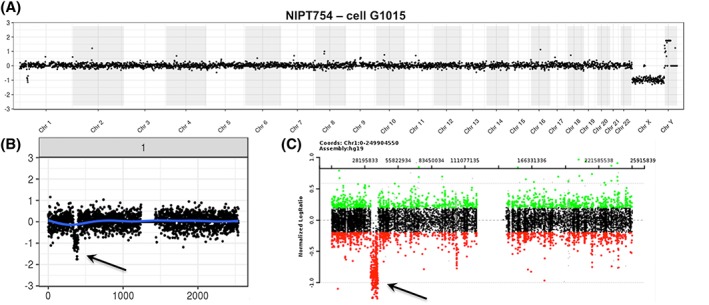
Detection of a 6.0‐Mb deletion of 1p35.1p34.3 in a male fetal cell**.** The whole genome plot for the cell‐based noninvasive prenatal testing (NIPT) result is shown in Panel A, demonstrating the comparison to a female fetal reference cell with the X and Y chromosome difference and showing a deletion on chromosome 1. The single chromosome 1 plot is shown in Panel B. The data are concordant with the chorionic villus sampling (CVS) chromosomal microarray (CMA) data shown in Panel C. The coordinates for the deletion from the array were chr1:33,058,933‐39,031,717 [Colour figure can be viewed at wileyonlinelibrary.com]

**Figure 5:**
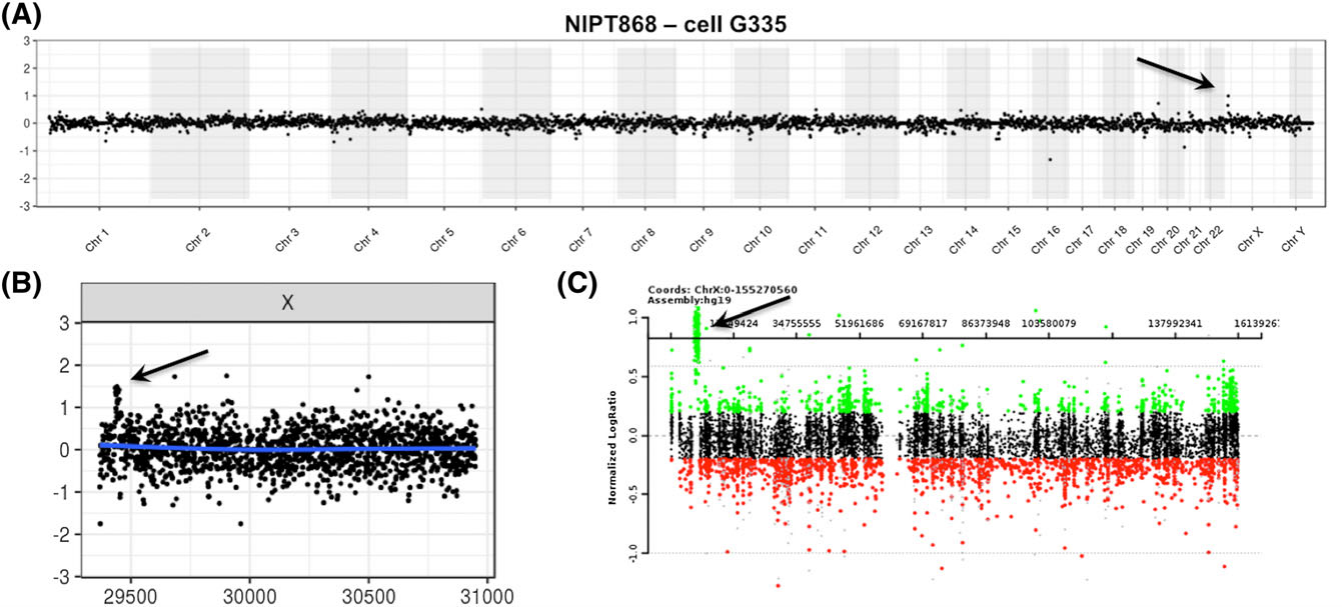
Detection of a 1.2‐Mb gain at Xp22.31. The cell‐based noninvasive prenatal testing (NIPT) whole genome plot with both the case and the control being male is shown in Panel A, with a suggestion of a duplication on Xp. The single chromosome plot in Panel B confirms the gain. Panel C shows the chromosomal microarray (CMA) result for the chorionic villus sampling (CVS) sample, and the array coordinates for the gain were chrX:6,866,449‐8,115,153 [Colour figure can be viewed at wileyonlinelibrary.com]

Additionally, there were five cases in this series with pathogenic subchromosomal deletions or duplications, with a size range from 153 kb to 18.9 Mb. For each of the four cases with abnormalities of greater than or equal to 1 Mb, fetal cells were isolated, and all CNVs were identified by our cell‐based NIPT assay (shown in Figures 2, 3, 4, 5) in concordance with the available microarray data from invasively obtained fetal samples. For the case with a 153‐kb intragenic *GPC3* deletion in Xq26.2q26.2, trophoblasts were identified but not sequenced for incidental reasons.

Figure 2 shows a case in which a female fetus carried an 18.9‐Mb loss of chromosome 4 (Wolf‐Hirschhorn region) and a 1.1‐Mb gain of chromosome 15, detected by CMA on an amniocentesis sample obtained at 33 weeks and 2 days. The maternal blood sample was collected at 34 weeks and 4 days (>1 week after procedure). Despite the advanced gestational age, three cells were isolated from maternal blood, for two of which the NGS data were consistent with the CMA result, while sequencing failed for the third cell.

In another case (Figure 3), the mother underwent both CVS and amniocentesis with subsequent CMA analysis, which revealed that the female fetus harbored a complex 15‐Mb gain of chromosome 16, containing three duplications, two triplications, and two quadruplications. The blood sample for our study, from which eight cells were isolated, was collected immediately before amniocentesis at 16 weeks and 2 days. Even though the single cell NGS analysis could not resolve the same detail in copy number changes, the cell‐based result clearly showed a similar result to the gain observed in the CMA data. The serum screening for this case, performed as part of the patient's clinical care, had indicated a one in five risk for Down syndrome and a first trimester NT of 2.8 mm.

Figure 4 illustrates the whole genome plot from a male fetus with a 6.0‐Mb loss of chromosome 1. The mother was of advanced maternal age and ultrasound examination showed fetal cystic hygroma. A CVS sample was collected at 13 weeks and 6 days, as well as a maternal blood sample 1 hour after procedure for cell‐based NIPT. As we could not recover any fetal trophoblastic cells from that sample, a redraw was done at 15 weeks and 4 days, and NGS data obtained from a trophoblast doublet from that sample were concordant with the CMA result obtained earlier.

Figure 5 shows a case in which a 1.2‐Mb gain of the X chromosome was detected in both the male fetus and his carrier mother. This sample was collected at 12 weeks and 1 day, before CVS. cfDNA‐based NIPT was done for this pregnancy as well but gave no reportable result because, as noted by the reported laboratory, “due to technical or sample‐related issues the data failed to meet the quality standards for interpretation (the patient reportedly has a large fibroid which is likely interfering with the analysis of the fetal DNA).”

### 3.4 Mosaicism

**Figure 6:**
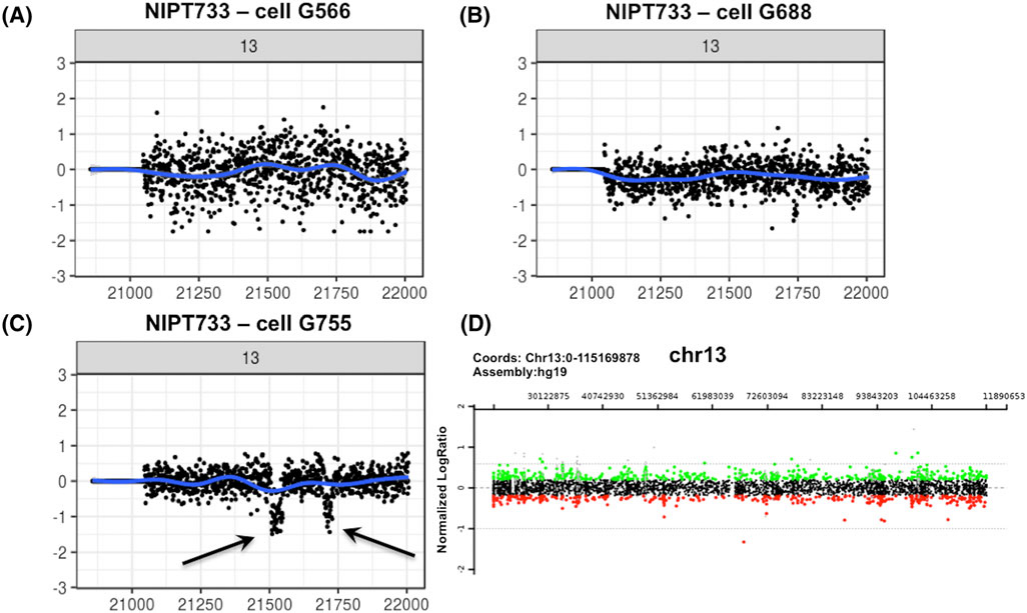
Detection of two mosaic deletions on chromosome 13. For this case, cfDNA‐based noninvasive prenatal testing (NIPT) indicated a possible partial or full monosomy 13, while chromosome analysis of chorionic villus sampling (CVS) tissue showed a normal male pregnancy. We retrieved three fetal trophoblastic cells of which two showed a normal chromosome 13 content (Panels A and B). The third fetal cell, however, showed two small deletions of 4 Mb and 2.6 Mb (Panel C). The normal CVS chromosomal microarray (CMA) result for chromosome 13 is shown in Panel D [Colour figure can be viewed at wileyonlinelibrary.com]

We encountered a case of possible confined placental mosaicism in this data set. Study subject NIPT733 first underwent cfDNA‐based NIPT, which indicated a “possible partial or full monosomy of chromosome 13.” The patient then underwent a CVS procedure with CMA analysis on DNA directly extracted from the CVS sample, without prior culture, which showed a normal result. Simultaneously, we enrolled this subject for our study and were able to isolate three fetal trophoblastic cells. One of those clearly showed two small losses (4 Mb and 2.6 Mb, respectively) on chromosome 13 (Figure 6), which we interpret as likely related to the cfDNA‐based NIPT result and not coincidental. The other cells did not show these deletions, implying that confined placental mosaicism led to the false positive cfDNA‐based NIPT result. This highlights the potential of cell‐based NIPT to demonstrate two different fetal genotypes in case of mosaicism, which cannot be achieved by cfDNA‐based testing.

### 3.5 Quality of single cell NGS data

**Figure 7:**
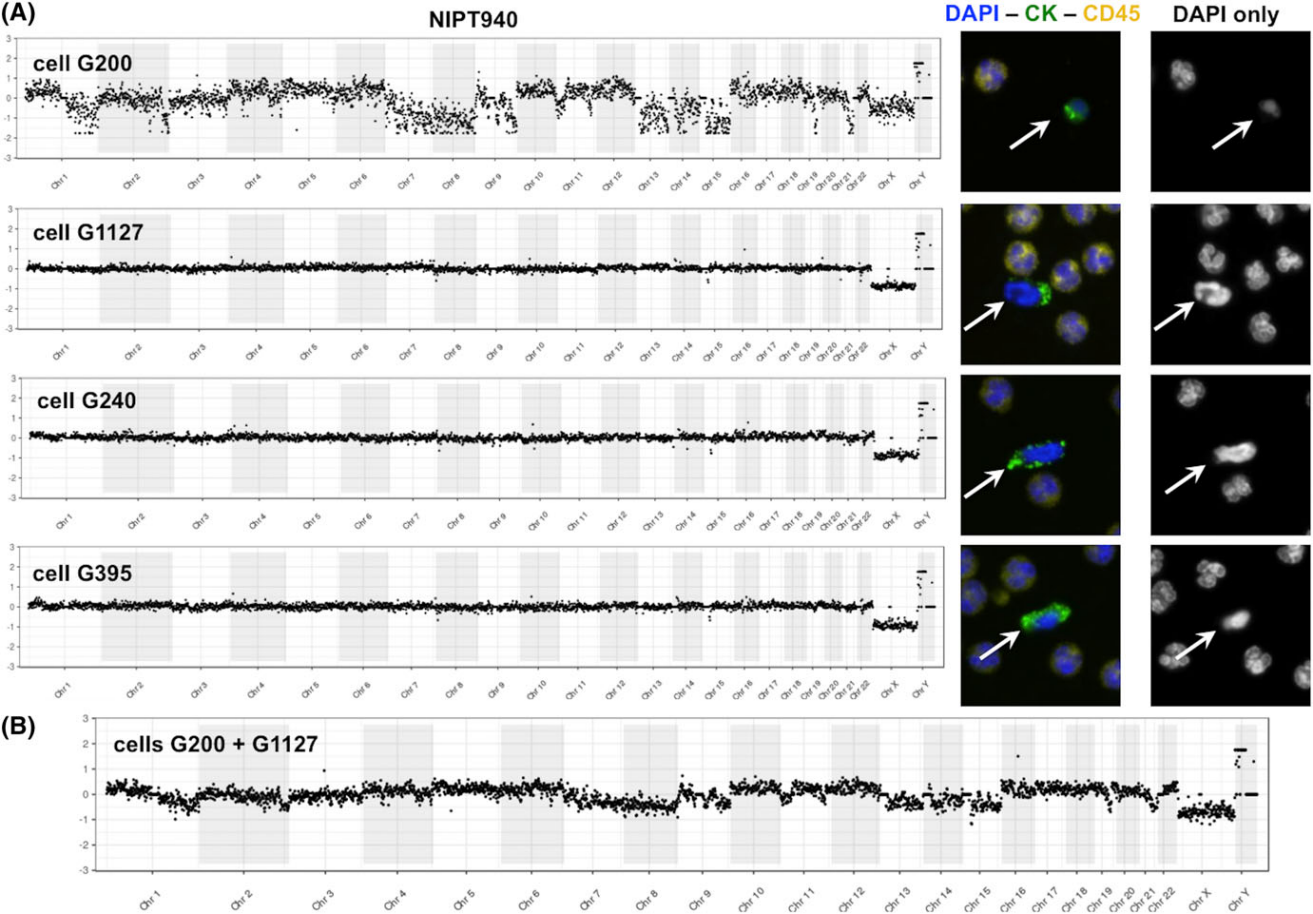
Segmental loss of copy number, including homozygous loss for entire chromosomes suggestive of apoptosis. The whole genome plots for four cells from a male fetus compared with female fetal reference are shown in Panel A. The plot for cell G200 is extremely noisy while that of the other three cells shows a normal male profile. The DAPI staining for this cell is faint compared with the other fetal (white arrows) and maternal cells. The highly segmental loss of copy number including homozygous loss for entire chromosomes or arms of chromosomes as seen in cell G200 is interpreted to represent apoptosis (see Section 4). Panel B shows the substantial impact of pooling the data of an apoptotic cell (G200) with the data of a cell of high quality (G1127): Even though the copy number changes seen for G200 are somewhat compensated by G1127, the overall profile remains useless for copy number variant (CNV) analysis nevertheless. This next‐generation sequencing (NGS) plot was generated by comparing this pool to a reference pool of two normal fetal cells [Colour figure can be viewed at wileyonlinelibrary.com]

In general, most isolated fetal cells yield NGS plots of adequate quality. Occasionally, however, we obtain NGS data of inferior quality, usually associated with fetal cells for which only a weak DAPI signal is observed during microscopic validation. Figure 7 illustrates the whole genome plots for four cells isolated from the same sample: cell G200 has a lower DAPI signal and shows extreme copy number loss going to near zero copies for multiple large chromosome segments (chr1, 9) or entire chromosomes (chr7, 8, 13, 15). As discussed below, this was interpreted as apoptosis based on prior experience on such profiles.^14^, ^15^, ^16^ Panel B in this figure illustrates the impact of pooling the data of an apoptotic (G200) and a high‐quality cell (G1127): although the pooled data show a less extreme profile compared with G200 by itself, the overall data are unusable for CNV analysis.

## 4 DISCUSSION

The results presented here further support the feasibility of cell‐based NIPT as a clinical test, in agreement with other publications.^9^, ^10^, ^11^, ^12^ Our data set represents the largest to date showing successful, reproducible analysis of single trophoblasts by NGS‐based CNV analysis with the goal of implementing a clinical cell‐based NIPT protocol. The major obstacle that remains is obtaining a consistent fetal cell yield for each sample. The goal of this study was primarily to develop and refine an optimal protocol; thus, the diagnostic results are still exploratory and preliminary, and further clinical validation is needed. We acknowledge that the protocol is still evolving. Our ultimate goal for clinical testing is to have excellent NGS data on three to five cells, but we have observed cases for which a single cell provided valuable information. Although 18.4% and 19.2% of the samples described here resulted in zero or one cell recovered, previously published reports on immunomagnetic bead enrichment indicate that fetal cell yield can be further increased.^10^, ^11^ Our own recent experience with a new protocol for positive trophoblast selection at present being explored in our lab, supports this (unpublished data). Any manipulation before or during the blood draw that could increase the number of trophoblasts in the maternal circulation, such as physical activity before blood collection, would also be helpful. However, exercise on a stationary bicycle provided a small increase in cell number.^17^ Our data suggest that trophoblast recovery is unlikely to be influenced by the maternal BMI that is known to be a cause for cfDNA‐based NIPT failure because of its lowering effect on the fetal fraction. The effect of a CVS/amniocentesis procedure on fetal cell yield for blood samples collected postprocedure has been described for fnRBCs.^18^ In our series the effect seems limited, but more samples are needed for statistical comparison.

Although the isolated fetal cells have a distinct cytokeratin staining pattern that has been used to validate their fetal origin,^11^ we think it is important to confirm fetal origin more conclusively when they are used for clinical, diagnostic cell‐based NIPT. Confirming fetal origin can be done by demonstrating the presence of an XY complement by NGS for male pregnancies. It is more challenging for female pregnancies, where genotyping is require to differentiate them from maternal cells that were incorrectly identified as likely fetal by microscopy.

Multiple pregnancies represent both a challenge and a potential strength for cell‐based NIPT. For example, for dizygous twins, it should be possible to obtain reliable independent data from both fetuses, assuming robust genotyping to distinguish cells from the two twins. In case of monozygous twins with identical CNV findings, testing a larger number of cells offers increased statistical confidence that both fetuses have been studied. For rare cases in which only one of monozygous twins is carrying a de novo CNV, the two CNV profiles should be distinguishable if enough cells are tested.

If more than one fetal cell is recovered from a maternal blood sample, there is the option to analyze the cells individually, or as a pool of cells. We prefer to analyze them as individual cells, even though this slightly increases WGA and sequencing costs. Occasionally, trophoblast clusters (≥2 fetal cells together) are identified, and we speculate that these may be recently released from the placenta and be daughter cells from a recent mitosis. When these clusters remain attached during the picking procedure, they are analyzed as one. As WGA product quality varies from cell to cell, we do not wish to risk compromising the NGS profile of one good quality cell by pooling with another cell of inferior quality. For instance, pooling cells G200 and G1127 shown in Figure 7 give an unusable result, although cell G1127 alone is useful. Since data from multiple single cells can also be pooled post NGS and data analysis, we recommend against pooling cells before WGA. Single trophoblast data can help to address mosaicism, as is suggested by our current and earlier data, and they are also preferred for multiple pregnancies.

Single cells with severe and often homozygous whole or segmental chromosome loss as illustrated in Figure 7 are interpreted to represent fetal cells undergoing apoptosis. Every apoptotic cell has its own unique pattern of copy number changes (often to zero copies), affecting multiple entire chromosomes. In contrast, a true CNV is seen in multiple cells of confirmed fetal origin from the same sample. Kolialexi et al summarized extensive evidence of apoptosis in fetal cells in the maternal circulation including much higher apoptosis rates in Down syndrome and Turner syndrome pregnancies.^14^ The apoptotic cells were suggested to be a source of cfDNA in mother's plasma. Bártová used FISH and TUNEL methods to demonstrate that “chromosomal territory segmentation precedes the formation of nuclear apoptotic bodies.”^15^ Particularly the chromosomal territory images shown in their publication^15^ lead us to interpret these fetal cells as apoptotic. Similar large homozygous deletions were described in cancer cells and termed chromazemic cells that might represent dying cells.^16^ No TUNEL or Annexin V staining was done in the study presented in this manuscript.

The new data reported here, combined with previously published results, show that a broad range of fetal chromosomal abnormalities, ranging from aneuploidies to subchromosomal gains or losses of greater than or equal to1 to 2 Mb in size can be detected. In all cases where useful NGS data were available except for one instance of presumed confined placental mosaicism, the findings in any fetal cell agreed with the findings after amniocentesis or CVS, and for cases where more than one fetal cells was scorable, the different cells showed similar NGS plots. At this stage of development, the processing time was lengthy, and the review of the NGS data was not blinded, although blinding is currently being used. For the detection of CNVs in fetal cells, it is desirable to have the highest resolution possible, so that also (de novo) microdeletions/duplications are detected, such as the 2 to 3 Mb 22q11.2 deletion causing DiGeorge syndrome, the 1.5‐Mb deletion causing Williams syndrome or the 1.5‐Mb CMT1A duplication. Reliable CNV detection at 220 kb resolution has been described in single tumor cells by Casasent, Schalck *,* and Gao^19^ and a new WGA method reported by Chen et al,^20^ reported the detection of micro CNVs as small as 100 kb. From a clinical perspective, we believe that CNV detection at 0.5 Mb is a reasonable short‐term goal for cell‐based NIPT.

## FUNDING INFORMATION

This work was funded by internal institutional funds at Baylor College of Medicine and corporate funds at RareCyte, Inc.

## CONFLICT OF INTEREST

The Houston authors are faculty and staff at Baylor College of Medicine (BCM), which is a partial owner of Baylor Genetics (BG), and/or are employee of, have advisory or lab director roles at BG. All Seattle authors are or were employees of RareCyte, Inc. RareCyte has patents licensed, owned or assigned that relate to the equipment that RareCyte manufactures but not to the methods described here.

## Supporting information

Data S1. Supporting information

Figure S1. Panel A shows two additional single cells for case NIPT864, in which the fetus had a 45,X complement, both compared to a normal female and normal male reference. Additionally, a loss in the polymorphic region of 15q11 for these two cells is shown in panel B. Panel C shows the NGS data for two additional cells of a female fetus with trisomy 18, and additional data for a male fetus with trisomy 21 is shown in panel D. Panel E illustrates two cells from a normal female fetus, which were both confirmed fetal by genotyping.

Table S1 Supporting information
