# Supplementary material for: Reliable detection of subchromosomal deletions and duplications using cell‐based noninvasive prenatal testing
Source: Prenat Diagn. 2018 Nov 19;38(13):1069–78. doi: 10.1002/pd.5377 (PMC6587831; doi:10.1002/pd.5377)
Supplement: Supplementary file 1 — Data S1. Supporting information [file PD-38-1069-s001.docx]

**Supplementary Methods**

*Sample collection*

Detailed information on each study subject and sample can be found in the Supplementary Table. A series of 125 samples were collected from 122 pregnant women. This includes three redraws, respectively for subjects NIPT733, NIPT754 and NIPT767. None of the samples have been described in a previous manuscript. All samples were stored or shipped overnight before processing, except for three samples for which the processing only could be started two days after collection due to sample transportation difficulties.

*Genomic DNA extraction*

Maternal gDNA, and paternal if a blood sample from the father was available, was extracted on a MagnaPure Compact platform (Roche), using 400 uL whole blood samples with the Nucleic Acid Isolation kit I (Roche). When paternal saliva samples were collected, the paternal gDNA was extracted on the same platform, using 500 uL saliva with the Nucleic Acid Isolation Large Volume kit I (Roche).

*Fetal sex determination*

Fetal cfDNA was extracted from the plasma after whole blood phase separation by centrifugation (10 min, 3,000xg, RT – carried out twice) on a MagnaPure Compact platform, using 500 uL of plasma with the Nucleic Acid Isolation Large Volume kit I. The eluted cfDNA was then used in a Y chromosome-specific quantitative PCR assay (quantification of single copy gene *SRY* and the multicopy sequence *DYS14* within the *TSPY* gene) on a LightCycler 480 platform (Roche). The PCR reaction conditions were: a hold of 3 min at 95°C, 49 cycles of 15 s at 95°C – 30 s at 64°C, followed by a final step of 30 s at 40°C. The used primers and TaqMan probes were previously described by Picchiassi *et al.*^1^ Each sample was run in triplicate, and several negative and positive controls were included per run.

*Trophoblast enrichment and isolation*

Individual fetal trophoblastic cells were isolated as described before by Breman *et al.*^2^. In short, a maternal WBC depletion was performed by a 20 min incubation with a RosetteSep CD45 and CD36 depletion cocktail (StemCell Technologies). The number of WBCs was measured beforehand with a QBC Autoread Plus Dry Hematology System (Drucker Diagnostics). Subsequently, 6 mL of blood was pipetted per AccuCyte Separation tube (RareCyte) and centrifuged (30 min, 3,000xg, RT). A first CyteSeal ring was applied below the band of nucleated cells formed around the float, after which the plasma was removed. Next, after a high-density displacement fluid was added to each tube, a second centrifugation step was performed (5 min, 1,000xg, RT) to lift the cells above the float, and a second CyteSeal was administered. Following a fixation step (60 uL 5% paraformaldehyde solution, 20 min incubation), a blocking/permeabilization solution was added (720 uL) and the cell suspension was subsequently stained for 1 h with a cocktail of DAPI, AF480-labeled anti-cytokeratin antibodies and a PE-labeled anti-CD45 antibody. Following incubation, the cell suspension was lifted into a collection tube, by means of consecutive layering steps with different higher density fluids and centrifugation (20 min at 1,000xg, RT). The tubes were taken out of the EpiCollector and PBS was added to the resulting cell pellets. All cell suspensions were counted with a hemocytometer, and stored at 4°C until scanning. The cell counts before and after processing were used to calculate the WBC depletion rate.

Each fraction was spread on CyteSlides at a density of 800,000 cells/well and scanned on the CyteScanner/CytePicker. After automated scanning, all detected candidates were manually validated for fetal cell identification by 40X magnification imaging. Candidates are evaluated based on three criteria: 1) positive cytokeratin (CK) staining in a specific pattern, 2) nuclear morphology and 3) absent staining for WBC marker CD45. The fetal CK pattern can vary from a vesicular pattern around the nucleus to a more diffuse staining. The vesicular or ‘bubble’ pattern is most common.^3^ The nuclear morphology of a fetal cell is generally round or more elongated, and has a smooth appearance, compared to maternal WBCs usually having a more lobed structure. All positively evaluated were picked as a single cell with a 40 um ceramic needle, and deposited in 2 uL of PBS in a 200 uL PCR tube, and stored at -80°C until further processing. All reagents and materials for processing and scanning were provided by RareCyte, unless specified otherwise.

*Whole genome amplification*

To obtain a sufficient amount of DNA material for downstream analysis, whole genome amplification (WGA) was performed separately for every single individual cell. The PicoPLEX WGA kit (Rubicon Genomics) was used, according to the manufacturer’s protocol. After the WGA process, an aliquot of each product was run on a 2 % agarose gel, to assess whether amplification was performed successfully. All well-amplified WGA products were purified with a Zymo DNA Clean and Concentrator-25 kit (Zymo Research) and eluted in 50 uL of nuclease-free water (Promega). The DNA yield and quality of the samples was measured on a NanoDrop (Thermo Fisher Scientific).

*Genotyping*

For confirmation of fetal origin, highly polymorphic genomic regions were analyzed in fetal single cell WGA products and parental gDNA samples by NGS. Ion Torrent libraries were constructed with a two-step tailed PCR approach, starting off with WGA products diluted to 25 ng/uL. In a first step, 15 amplicons of interest were amplified by multiplex PCR (initial 30 s at 98°C, 13 cycles of 10 s at 98°C – 15 s at 68°C – 30 s at 72°C, final 2 min step at 72°C and hold at 4°C), targeting HLA-A and HLA-B loci, specific loci on chromosomes 1, 2, 7, 9, 11, 14, 15, 16 and 21, and the Y chromosome.

A second round of PCR was performed for introducing barcodes and sequence adaptors: the used forward primer contained a 30 nt A adaptor for sequencing and a 10 nt Ion Xpress barcode, while the reverse primer includes a 23 nt P1 adaptor. The following PCR conditions were applied: 30 s at 98°C, 25 cycles of 10 s at 98°C – 15 s at 68°C – 30 s at 72°C, followed by a final extension 2 min at 72°C and a final hold at 4°C.

The resulting PCR products were run on a 2 % agarose gel and purified with Agencourt AMPure XP Reagent (Beckman Coulter) according to the manufacturer’s instructions, after which the concentration was determined with a Bioanalyzer 2100 instrument (Agilent). Where necessary, samples were diluted to obtain an equimolar library across all samples to be pooled for sequencing. Further library preparation was performed with the Ion PGM Template OT2 200 kit according to the manual (Thermo Fisher Scientific), followed by single-end sequencing with the Ion PGM Sequencing 200 kit v2 (Thermo Fisher Scientific) on an Ion Torrent platform (Thermo Fisher Scientific).

After sequencing, the resulting reads were aligned to the reference target genome, and all candidate single nucleotide polymorphisms (SNP) were extracted. SNP-only reads were generated using an in house-developed script and compared with the parental gDNA. A cell is scored as being fetal when the corresponding WGA products shows two or more polymorphic alleles that are not present in the maternal gDNA; one such allele is scored as likely fetal.

*CNV analysis*

For CNV analysis, 300 ng of WGA product in a final volume of 50 uL was sheared using the Covaris E220 System, with a target size of 200 to 700 bp. Fragment size was checked afterwards by running the samples on gel. Subsequently, end repair was performed (New England Biolabs reagents), followed by a SPRI bead (Beckman Coulter) cleanup step. A-tailing (NEB reagents) was done, followed by another cleanup. In a next step, Illumina adaptors were ligated and samples were purified again, after which the SPRI beads were removed. A round of PCR with specific Illumina primers was performed, after which the products were cleaned up with Agencourt AMPure XP Reagent (Beckman Coulter) according to the manufacturer’s instructions and the concentration was determined with a Bioanalyzer 2100 instrument (Agilent). Subsequent paired-end, whole genome sequencing was performed on a HiSeq platform (Illumina), aiming for about 5 x 10^6^ unique reads per cell with 100 bp read length, giving a genome coverage of about 0.3X.

Sequence files were mapped against the human genome (hg19) using BWA-MEM (v. 0.7.15). The bedtools’ (v. 2.25.0) function multicov generates coverage counts, which were then interpreted, compared on a one-to-one basis, and visualized using an in house-developed web tool. This generates a view (1 Mb bin size whole genome plot) used for the detection of copy number abnormalities. The quality of each cell was scored manually.

**References**

1. Picchiassi E, Coata G, Fanetti A, et al. The best approach for early prediction of fetal gender by using free fetal DNA from maternal plasma. Prenat Diagn 2008;28(6):525–30.

2. Breman AM, Chow JC, U’Ren L, et al. Evidence for feasibility of fetal trophoblastic cell-based noninvasive prenatal testing. Prenat Diagn 2016;36(11):1009–19.

3. Kølvraa S, Singh R, Normand EA, et al. Genome-wide copy number analysis on DNA from fetal cells isolated from the blood of pregnant women. Prenat Diagn 2016;36(12):1127–34.
