# Supplementary figures and images for: Reliable detection of subchromosomal deletions and duplications using cell‐based noninvasive prenatal testing
Source: Prenat Diagn. 2018 Nov 19;38(13):1069–78. doi: 10.1002/pd.5377 (PMC6587831; doi:10.1002/pd.5377)

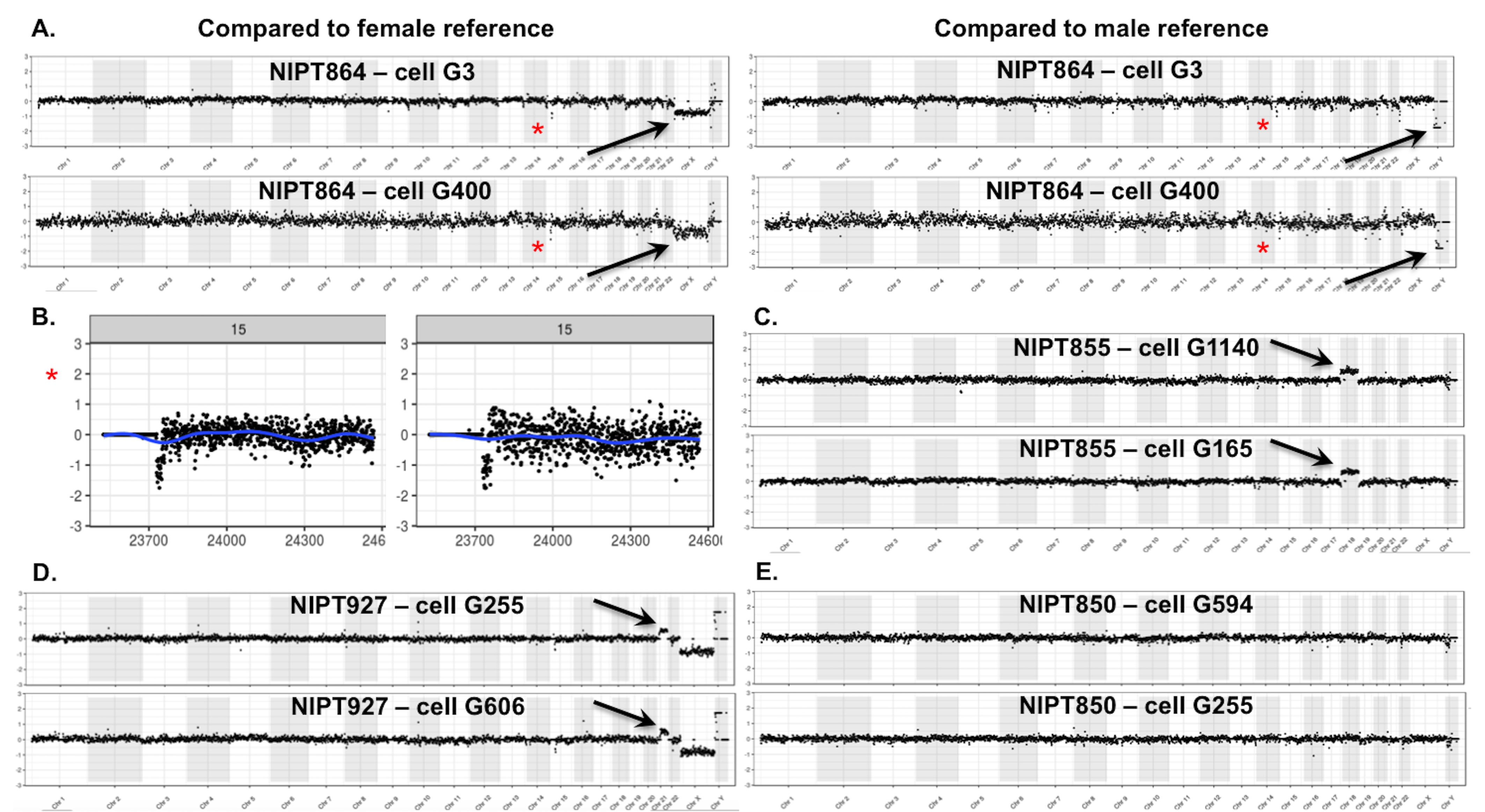

Supplement: Supplementary file 2 — Figure S1. Panel A shows two additional single cells for case NIPT864, in which the fetus had a 45,X complement, both compared to a normal female and normal male reference. Additionally, a loss in the polymorphic region of 15q11 for these two cells is shown in panel B. Panel C shows the NGS data for two additional cells of a female fetus with trisomy 18, and additional data for a male fetus with trisomy 21 is shown in panel D. Panel E illustrates two cells from a normal female fetus, which were both confirmed fetal by genotyping. [file PD-38-1069-s002.png]
